# A novel full-human CD22-CAR T cell therapy with potent activity against CD22^low^ B-ALL

**DOI:** 10.1038/s41408-021-00465-9

**Published:** 2021-04-10

**Authors:** Yue Tan, Haodong Cai, Chuo Li, Biping Deng, Weiliang Song, Zhuojun Ling, Guang Hu, Yongkun Yang, Panpan Niu, Guangrong Meng, Wei Cheng, Jinlong Xu, Jiajia Duan, Zelin Wang, Xinjian Yu, Xiaoming Feng, Jianfeng Zhou, Jing Pan

**Affiliations:** 1 State Key Laboratory of Experimental Hematology, Boren Biotherapy Translational Laboratory, Boren Clinical Translational Center, Department of Hematology, Beijing Boren Hospital, Beijing, 100070 China; 2 State Key Laboratory of Experimental Hematology, National Clinical Research Center for Blood Diseases, Institute of Hematology & Blood Diseases Hospital, Chinese Academy of Medical Sciences & Peking Union Medical College, Tianjin, 300020 China; 3 Department of Hematology, Tongji Hospital, Tongji Medical College, Huazhong University of Science and Technology, Wuhan, 430030 China; 4 Cytology Laboratory, Beijing Boren Hospital, Beijing, 100070 China; 5 Department of Hematology, Beijing Boren Hospital, Beijing, 100070 China; 6 Nanjing Iaso Biotherapeutics Co. Ltd, Nanjing, 210000 China; 7 Medical Laboratory, Beijing Boren Hospital, Beijing, 100070 China; 8 Central Laboratory, Fujian Medical University Union Hospital, Fuzhou, 350001 China

**Keywords:** Phase I trials, Immunotherapy, Acute lymphocytic leukaemia

Dear Editor,

CD22-targeted chimeric antigen receptor (CD22-CAR) T cells have been proven to be effective in treating patients with B acute lymphoblastic leukemia (B-ALL) who were unsuitable to receive CD19-CAR T cell therapy^1–3^. However, a considerable proportion of patients still relapsed after CD22-CAR T cell therapy^3,4^, with diminished or decreased levels of CD22 expression on blasts. For patients who had not completely lost CD22 expression on blasts, CD22-CAR T cell re-treatment may apply as a salvage regimen. But unfortunately, in a previous trial on a humanized CD22-CAR (termed CD22-CAR^YK002^) T cell therapy, a second CD22-CAR^YK002^ infusion produced no or suboptimal anti-leukemia response and CAR T cell expansion (Table S1 and Fig. S1). Because the immunogenicity of humanized antibodies or CARs has been suggested in previous studies^5–8^, the poor effect of the second CD22-CAR^YK002^ infusion might be due to the patient’s immune response against CD22-CAR^YK002^ transgene. On the other hand, CD22 downregulation might also represent a mechanism of resistance to a second CAR T-cell therapy^3^. Therefore, a new CD22-CAR with no cross immunogenicity with CD22-CAR^YK002^, and with strong activity against CD22^low^ cells, may be effective for the second treatment of patients who failed from previous CD22-CAR^YK002^ T cell therapy. Furthermore, since the immunogenicity of full-human antibodies tends to be reduced compared with humanized or chimeric constructs^9–11^, the usage of fully human-derived CAR constructs might be a better strategy, which can further lower the risk of developing immune responses against the secondarily infused CAR T cells.

This study aims to develop a new CD22-CAR construct with low immunogenicity and potent activity for treating B-ALL patients who failed from prior CD22-CAR T cell therapies. Full-human anti-CD22 single chain fragment variant(scFv)s were screened from a full-human scFv yeast display library. The screened anti-CD22 scFvs were fused to the intracellular 4-1BB co-stimulatory and CD3ζ signaling domains to create a panel of CD22-BBz variants (Fig. 1a). The activities of the different CD22-BBz variants were tested with NFAT reporter assay in Jurkat cells in response to CD22^high^ Raji, CD22^low^ JVM-2, and CD22^-^ K562 cells (Fig. 1b), and CD22-BBz 80, 27, and 36 were identified as constructs that could transmit strong antigen-specific activation signals in T cells (Figs. 1c and S2). We then evaluated the effector function of different CD22-CAR variants via CD107a degranulation and cytotoxicity assay and identified CD22-BBz 80 with superior effector activity against CD22^low^ target cells (Fig. 1d, e). The membrane proteome array (MPA) showed that CD22-BBz 80 had a high specificity to the target antigen (Fig. 1f). The in vivo anti-leukemia effect of CD22-BBz 80 was confirmed in NPG mice injected with 1 × 10^6^ Nalm6-Luc cells (Fig. S3). Thus, CD22-BBz 80 (termed CD22-CAR^FH80^) was identified to have potent and antigen-specific anti-leukemia activity and was used in the subsequent clinical study.

**Fig. 1:**
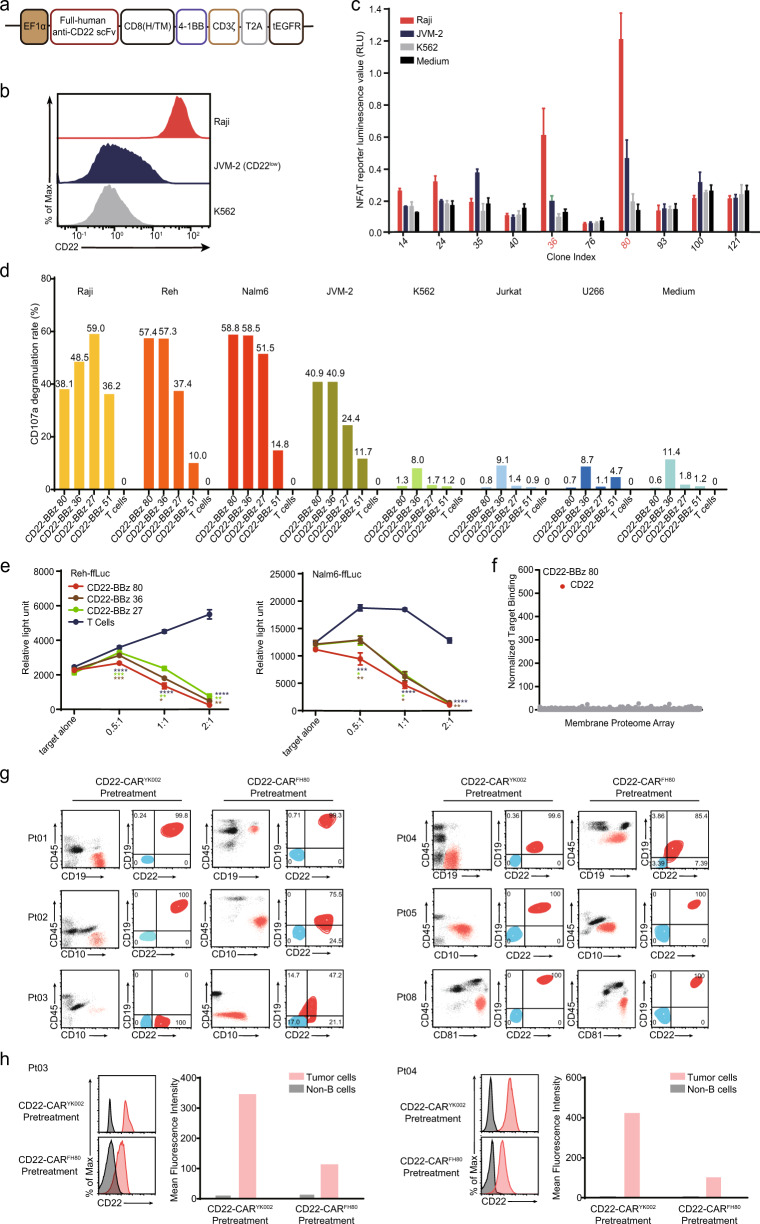
Development of a novel full-human CD22-CAR^FH80^ construct and leukemic CD19/CD22 expression in patients before enrolling in CD22-CAR^FH80^ therapy. **a** Schematic of the recombinant lentiviral vectors encoding the full-human CD22-BBz variants. The expression of CAR transgene is under the control of the elongation factor 1α promoter. CD8 (H/TM), CD8α hinge and transmembrane domains; EGFR, epidermal growth factor receptor; T2A, Thoseaasigna virus 2A. **b** Histogram of CD22 expression on Raji, JVM-2, and K562 cells, determined by flow cytometry. **c** Nuclear factor of activated T cells (NFAT) reporter assay in T cells transduced with different CD22-BBz variants, after co-culturing with CD22^high^ Raji, CD22^low^ JVM-2, and CD22^-^ K562 cells, or cultured in medium alone in triplicates. **d** Bar chart showing CD107a expression in specific CD22-BBz T cell clones after co-culturing with CD22^high^ Raji, Nalm6, Reh cells, CD22^low^ JVM-2, CD22^−^ K562, Jurkat, and U266 cells, as determined by flow cytometry. CD107a degranulation rates were calculated as the percentages of CD107a^+^ cells among CAR^+^CD8^+^ T cells and indicated above each bar. **e** Cytolytic effects of specific CD22-BBz T cell clones against Nalm6 and Reh cells during co-culturing in triplicates at E:T ratios of 2:1, 1:1, 0.5:1, and 0:1. % specific lysis = (spontaneous relative light unit (RLU) − test RLU)/(spontaneous death RLU) × 100. A two-tailed, unpaired two-sample *t*-test was used for statistical analysis. The brown, green, and blue asterisks indicate the comparison of the cytolytic effects of CD22-BBz 80 with those of CD22-BBz 36, CD22-BBz 27, or control T cells, respectively. *P < 0.05, **P < 0.01, ***P < 0.001, ****P < 0.0001. **f** Membrane proteome array for CD22-BBz. **g** Dot plots showing the proportions of blasts (red) and non-tumor cells (black) among all mononuclear cells from patients at the indicated time points, and contour plots showing CD22 and CD19 expression on blasts (red) before CD22-CAR^YK002^ and CD22-CAR^FH80^ T cell therapy, determined by flow cytometry with a population of CD22-negative non-B cells with similar cell size in the same staining tube (blue) as a negative control for evaluating CD22 expression level. The blasts were defined based on the combined analysis of multiple markers. The number represents the proportion of blasts (red) in the four quadrants. **h** Histogram and mean fluorescence intensity of CD22 cell-surface expression in patients 03 and 04.

A single-center, open-label, phase I clinical trial was conducted to evaluate the safety and efficacy of CD22-CAR^FH80^ T cells in 8 children with CD22^+^ or CD22^low^ B-ALL who failed in prior CD19 and CD22-CAR therapies. The median age was 9 (range, 5–16) years. Six patients (75%) had hematological relapses as confirmed by bone marrow morphology. One patient (pt 01) had persistent positive measurable residual disease (MRD^+^) detected by flow cytometry and the other patient (Pt 06) was MRD^−^ in bone marrow but had diffused extramedullary disease (Table S2). Four patients (50%; Pt 01, 04, 06, and 08) had previously undergone allogeneic hematopoietic stem cell transplantation (allo-HSCT 3 of 4 once, 1of 4 twice). All patients had received at least two lines of treatments including chemotherapies, allo-HSCT, CD19, or CD22 CAR T therapies. All patients (100%) failed from prior versions of CD22-CAR therapies (two refractory, six relapsed Fig. S4 and Table S2**)**. The CD22 expression level on blasts was not obviously reduced in six patients after prior CD22-CAR^YK002^ therapies, while patients 03 and 04 displayed lower CD22 expression on blasts than that before the CD22-CAR^YK002^ treatments (Fig. 1g, h). After receiving lymphodepleting chemotherapy with fludarabine (30 mg/m^2^/day) and cyclophosphamide (250 mg/m^2^/day), all patients received 1 (range, 0.68 to 9.4) × 10^6^ CD22-CAR^FH80^ T cells per kilogram body weight (/kg) (Table S3**)**.

Seven (87.5%) of eight patients had a response to CD22-CAR^FH80^ T cells. Six patients (75%) achieved MRD^-^ complete remission, including two (Pt 03, 04) with a low level of CD22 expression at enrollment; one patient (Pt 06) achieved partial remission in his extramedullary disease, but unfortunately, she succumbed to the infection on day 42 after infusion (Fig. 2a, b and Fig. S5). One non-response patient achieved remission after receiving inotuzumab, but died due to transplantation complication at 5 months. The six remission patients were followed up with a median time of 6 months. Of the three patients (Pt 02, 04, and 08) who received no further treatment, two (Pt 04 and 08) remained in remission for 9 and 5 months, while one (Pt 02) had a CD22^−^ relapse and died of tumor progression 6 months after infusion. Three patients (Pt 01, 03, and 07) were bridged to HSCT as consolidation after CAR T cell infusion (the donors and pre-conditioning regimen are detailed in Table S4), and among them, two (Pt 01 and 07) remained in remission 10 and 5 months after infusion, and one (Pt 03) had a relapse with a mixture of CD22^low^ and CD22^−^ blasts 3 months after HSCT (Fig. S6).

**Fig. 2:**
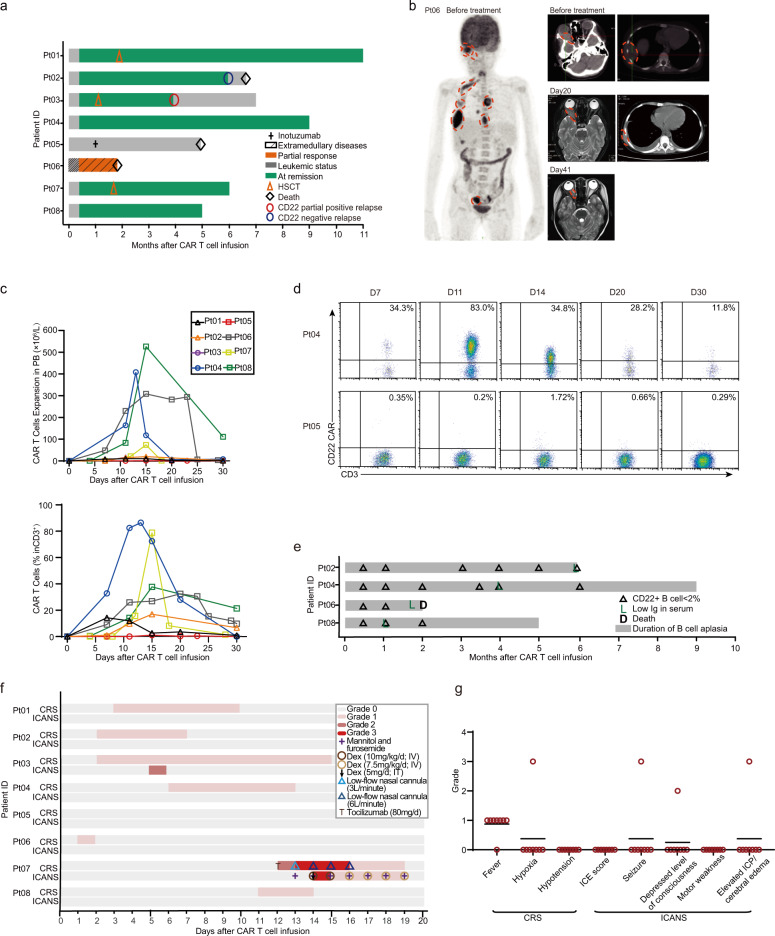
Clinical efficacy and safety of CD22-CAR^FH80^ T cell therapy. **a** Swimmer plot showing the clinical responses, response duration, and outcome in individual patients treated with CD22-CAR^FH80^ T cells. HSCT, hematopoietic stem cell transplantation. **b** Images showing the retreatment extramedullary disease detected by position emission tomography (PET), the reduction of intracranial mass detected by magnetic resonance imaging (MRI), and the reduction of chest wall mass detected by computed tomography (CT) during CAR T cell treatment in patient 6. The red dotted circle indicates the border of a leukemia mass. **c** Absolute number of CAR T cells (above panel) and the percentage of CAR T cells among CD3^+^ T cells (below panel) in the peripheral blood of eight patients who received CD22-CAR^FH80^ T cell therapy. **d** Representative dot plot, detected by flow cytometry, showing the presence of CD22-CAR^FH80^ T cells among CD3^+^ T cells in the peripheral blood of two patients, including Pt 4 who achieved complete remission and Pt 5 who did not respond to the therapy. **e** Swimmer plot showing the duration of B cell aplasia in the bone marrow and hypogammaglobulinemia in four patients who achieved complete remission but received no further treatments. Serum immunoglobulins and bone marrow non-malignant B cells were determined by immunoturbidimetry and flow cytometry, respectively. **f** Swimmer plot showing the grade, duration, and management CRS and ICANS in each patient. The color in the swimmer lane indicates the existence of a specific grade of CRS or ICANS at a specific time point during CAR T cell treatment. IT intrathecal injection IV intravenous injection CRS Cytokine-release syndrome ICANS immune effector cell-associated neurotoxicity syndrome. **g** Symptoms of CRS and ICANS in individual patients during CD22-CAR^FH80^ T cell therapy. Horizontal lines indicate mean values of the grade of specific symptoms.

The expansion of CD22-CAR^FH80^ T cells peaked from days 11 to15 after infusion with a median count of 19.8 (range, 1.01–408) × 10^6^/L and a median percentage of 30 (range, 1–45) % among CD3^+^ T cells in the blood (Fig. 2c, d). The non-responding patient (Pt 05) had a significantly lower peak CAR T cell expansion (1.01 × 10^6^/L; 1% among CD3^+^ T cells) than other patients, despite her cultured CAR T cell viability (76.5%) transduction efficiency (44%) and infused dose (3.49 × 10^6^/kg) being at intermediate to high levels among all patients (Table S5).

B cell aplasia and hypogammaglobulinemia could be used as a measurement of the active surveillance of CAR T cells^12^. The three patients who achieved remission and received no further treatments all exhibited B cell aplasia and hypogammaglobulinemia until the observation end point (Fig. 2e), suggesting a prolonged persistence of CAR T cells.

CRS occurred in 7/8 (87.5%) patients, including six with grade 1 CRS (75%) and 1 with grade 3 CRS (12.5%). The median time to onset was 3 days (range, 1–12 days), and the median duration was 10 days (range, 2–19 days). The non-responding patient exhibited no signs of CRS, in accordance with his minimal CAR T cell expansion. Neurologic toxicities occurred in 2/8 (25%) patients, including 1 with grade 2 (12.5%) and 1 with grade 3 (12.5%). The detailed manifestations and managements of CRS, ICANS, and other toxicities suspected to be related to CAR T cells are shown in Fig. 2f, g and Table S6. We further compared the severity of CRS and ICANS between CD22-CAR^FH80^ and the prior CD22-CAR^YK002^ therapies in the same individual patients but found no significant difference (*P* = 0.414 and 0.285, Fig. S7). Serum cytokines indicative of systemic inflammation were detailed in Fig. S8. Most patients had dramatic increases in IL-6, ferritin, and sCD25, while only a small proportion of the patients had an obvious increase in TNF-α and IL-10. The patient (Pt 07) who developed grade 3 CRS and ICANS showed the highest peak levels of IL-6, ferritin, and sCD25.

In this study, we developed a novel full-human CD22-CAR^FH80^ construct with superior activity against CD22^low^ target cells. In seven of eight B-ALL patients who were refractory or relapsed after previous CD19- and CD22-CAR T cell therapies, CD22-CAR^FH80^ T cell therapy exhibited potent anti-tumor activity with a manageable safety profile. The high response rate with CD22-CAR^FH80^ therapy implicates that there is no overt cross immunogenicity between CD22-CAR^FH80^ and prior infused CD22-CAR^YK002^ transgenes. Markedly, CD22-CAR^FH80^ therapy was even effective in two patients with CD22^low^ blasts, while in the two patients who relapsed after CD22-CAR^FH80^ therapy CD22^−^ blasts were majorly present, indicating that CD22^low^ leukemia cells can be efficiently eliminated by CD22-CAR^FH80^ T cells. Three patients without HSCT consolidation remained in B cell aplasia and hypogammaglobulinemia until the cut-off date, indicating the continued presence of CD22-CAR^FH80^ T cells, but the follow-up time is not long enough, and the immunogenicity and long-term persistent capability of CD22-CAR^FH80^ need to be further investigated. Our previous version of CD22-CAR^YK002^ was also very efficient in inducing remission in B-ALL^2^. Whether CD22-CAR^FH80^ outperforms CD22-CAR^YK002^ in persistence or long-term efficacy still warrants future study. In addition, a CD22-CAR containing a full-human scFv, termed m971, generated from a human phage library has been reported to produce convincing activity in B-ALL patients in a phase I trial^3^. The specific property and value of different CD22-CAR versions need to be further addressed in future studies. Although future phase 2/3 trials are needed to verify and optimize CD22-CAR^FH80^ therapy, our study indicates an important value of CD22-CAR^FH80^ as a salvage regimen for advanced B-ALL cases that are refractory to prior versions of CD22-CAR therapies.

## Supplementary information

Supplemental materials

## Data Availability

All data associated with this study are present in the paper or the Supplementary Materials. Any data related to the clinical trial can be emailed to the corresponding author.
