# Supplementary material for: A novel full-human CD22-CAR T cell therapy with potent activity against CD22low B-ALL
Source: Blood Cancer J. 2021 Apr 10;11(4):71. doi: 10.1038/s41408-021-00465-9 (PMC8036232; doi:10.1038/s41408-021-00465-9)
Supplement: Supplementary file 1 — Supplemental materials [file 41408_2021_465_MOESM1_ESM.pdf]

Supplemental Materials for

A novel full-human CD22-CAR T cell therapy with potent activity against $CD22^{low}$ B-ALL

Yue Tan, Haodong Cai, Chuo Li, Biping Deng, Weiliang Song, Zhuojun Ling, Guang Hu, Yongkun Yang, Panpan Niu, Guangrong Meng, Wei Cheng, Jinlong Xu, Jiajia Duan, Zelin Wang, Xinjian Yu, Xiaoming Feng, Jianfeng Zhou, Jing Pan

Table of Contents	Page
**Supplemental methods**	1
**Supplemental results**	7
**Supplemental tables**	
Supplemental Table 1: Baseline Characteristics of Patients Treated with CD22-CAR^{YK002} T cells	10
Supplemental Table 2: Baseline Characteristics of Patients Treated with CD22-CAR^{FH80} T cells	11
Supplemental Table 3: Characteristics of Infused CD22-CAR^{FH80} T Cell Product	12
Supplemental Table 4: The Characteristics and Donor Types of Patients Who Received HSCT after CD22-CAR^{FH80} T Therapy	13
Supplemental Table 5: CD22-CAR^{FH80} T Cell Expansion, Clinical Response and Toxicity	14
Supplemental Table 6: Adverse Events Summary During CD22-CAR^{FH80} T Cell Therapy	15
**Supplemental figures**	
Supplemental Figure 1: CD22-CAR^{YK002} T-cell second treatment was ineffective in 4 patients who relapsed after primary CD22-CAR^{YK002} therapies	16
Supplemental Figure 2: Nuclear Factor of Activated T cells (NFAT) Reporter Gene Assay	17
Supplemental Figure 3: CD22-CAR^{FH80} T Cells show superior anti-leukemia activity in vivo	18
Supplemental Figure 4: Treatment history in 8 patients before enrolling in CD22-CAR^{FH80} therapy	19
Supplemental Figure 5: MRI Scans of Intracranial Infection in Pt 06	20
Supplemental Figure 6: Clinical response and change of CD22 expression in response to CD22-CAR^{FH80} T cell therapy	21
Supplemental Figure 7: The CRS and ICANS Grade Comparison between CD22-CAR^{FH80} and CD22-CAR^{YK002} Therapy	22
Supplemental Figure 8: Elevation in serum cytokine levels after CD22-CAR^{FH80} T cell infusion	23
**References**	24

Supplemental Methods

Protocols of clinical trials

1. Phase I study of autologous fully humanized CD22-CAR T cells in the treatment of refractory or relapsed acute B lymphoblastic leukemia.

We conducted a phase I clinical trial (ClinicalTrials#: ChiCTR2000028793) of autologous anti-CD22 (full-human derived) chimeric antigen receptor T cells treating refractory or relapsed B acute lymphoblastic leukemia in Beijing Boren Hospital. The study was approved by the institutional review board of Beijing Boren Hospital, and informed consent was obtained in accordance with the Declaration of Helsinki. All patients agreed that their materials would be used for medical research. All these patients matched the diagnostic criteria for r/r B-ALL according to the WHO classification and completed morphological evaluation, immunophenotype analysis by flow cytometry (FCM), cytogenetic analysis by routine G-banding karyotype analysis and leukemia fusion gene screening by multiplex nested reverse transcriptase-polymerase chain reaction (PCR). Extramedullary diseases (EMDs) were confirmed CD22^{+} by FCM and evaluated by positron emission tomography/computed tomography (PET/CT), CT, magnetic resonance imaging (MRI) or ultrasonography. Enrolled patients received CD22 between January 9, 2020 and July 16, 2020 and were evaluated for responses and adverse effects. After CAR T cell infusion, clinical outcomes including overall survival (OS), Leukemia-free survival (LFS), adverse effects and relapse were evaluated up to date as of December 1st, 2020.

1.1 Inclusion and exclusion criteria for enrolled patients

1.1.1 Inclusion Criteria

1.1.1.1 Patients who were diagnosed as primary refractory or relapsed B-ALL[1]; Patients who haven't achieved a CR or relapsed after one regimen of chemotherapy, CD19 CAR T-cell therapy or humanized CD22 CAR T cell therapy; Patients had CD22 positive on leukemia blasts by FCM;

1.1.1.2 Age from 1 year old to 18 years old;

1.1.1.3 Candidates over 8 years old need to be sufficiently conscious and able to sign the treatment consent form and voluntary consent form;

1.1.1.4 Children candidates can be recruited after the legal guardian or patient advocate has signed the treatment consent form and voluntary consent form;

1.1.2 Exclusion criteria

1.1.2.1 Intercranial hypertension or unconscious;

1.1.2.2 Acute heart failure or severe arrhythmia;

1.1.2.3 Acute respiratory failure;

1.1.2.4 Other types of malignanttumors;

1.1.2.5 Diffuse intravascularcoagulation;

1.1.2.6 Serum creatinine and/or blood urea nitrogen over 1.5 times than normal range;

1.1.2.7 Sepsis or other uncontrolled infection;

1.1.2.8 Uncontrolled diabetes mellitus;

1.1.2.9 Severe psychological disorder;

1.1.2.10 Obvious cranial lesions with cranial MRI;

1.1.2.11 More than 20 counts/ul leukemic cells in cerebrospinal fluid;

1.1.2.12 More than 30% leukemic cells in blood;

1.1.2.13 Stage III WHO/ECOG score;

1.1.2.14 Organ recipients;

1.1.2.15 Pregnant or breastfeeding;

1.1.2.16 Active, uncontrolled infection, including hepatitis B, hepatitis C orhuman immunodeficiency virus (HIV);

1.2 Outcome measures

1.2.1 Primary Outcome measures

1.2.1.1 Identification of related adverse events. [Time Frame: 4 weeks after CAR T cellinfusion]

1.2.2 Secondary Outcome Measures

1.2.2.1 Identification of the efficacy [Time Frame: 4 weeks after CAR T cell infusion] Assessment of CR with incomplete blood count recovery, Partial response (PR) and Stable Disease.

1.2.2.2 In vivo persistence/expansion of infused CAR T cell [Time Frame: Up to 1 year] Detection of infused CAR T cell in the peripheral blood, bone marrow and cerebrospinal fluid. Detection of B cell aplasia and hypogammaglobulinemia.

1.2.2.3 Identification of related long-term adverse events. [Time Frame: Up to 1year]

2. Phase I study of autologous humanized anti-CD22 chimeric antigen receptor T cells treating refractory or relapsed B acute lymphoblastic leukemia.

We conducted a phase I clinical trial (ClinicalTrials#: ChiCTR-OIC-17013523) of autologous humanized anti-CD22 chimeric antigen receptor T cells treating refractory or relapsed B acute lymphoblastic leukemia in Beijing Boren Hospital. The study was approved by the institutional review board of Beijing Boren Hospital, and informed consent was obtained in accordance with the Declaration of Helsinki. All these patients matched the diagnostic criteria for r/r B-ALL according to the WHO classification and completed morphological evaluation, immunophenotype analysis by FCM, cytogenetic analysis by routine G-banding karyotype analysis and leukemia fusion gene screening by multiplex nested reverse transcriptase-polymerase chain reaction. EMDs were confirmed CD22+ by FCM and evaluated by PET/CT, CT, MRI or ultrasonography. Enrolled patients received CD22 between July 6, 2017 and May 9, 2018 and were evaluated for responses and adverse effects. After CAR T-cell infusion, clinical outcomes including OS, LFS, adverse effects and relapse were evaluated up to date as of May 1st, 2020.

2.1 Inclusion and exclusion criteria for enrolled patients

2.1.1 Inclusion Criteria

2.1.1.1 Patients who were diagnosed as primary refractory or relapsed B-ALL[1]; Relapsed patients who haven't achieved a CR after one regimen of chemotherapy, even CD19 CAR T-cell therapy; Patients had CD22 positive on leukemia blasts by FCM (>95% of CD22positive);

2.1.1.2 Age from 1 year old to 70 years old;

2.1.1.3 Candidates over 18 years old need to be sufficiently conscious and able to sign the treatment consent form and voluntary consent form;

2.1.1.4 Children candidates can be recruited after the legal guardian or patient advocate has signed the treatment consent form and voluntary consent form;

2.1.2 Exclusion criteria

2.1.2.1 Intercranial hypertension or unconscious;

2.1.2.2 Acute heart failure or severe arrhythmia;

2.1.2.3 Acute respiratory failure;

2.1.2.4 Other types of malignanttumors;

2.1.2.5 Diffuse intravascularcoagulation;

2.1.2.6 Serum creatinine and/or blood urea nitrogen over 1.5 times than normal range;

2.1.2.7 Sepsis or other uncontrolled infection;

2.1.2.8 Uncontrolled diabetesmellitus;

2.1.2.9 Severe psychologicaldisorder;

2.1.2.10 Obvious cranial lesions with cranial MRI;

2.1.2.11 More than 20 counts/ul leukemic cells in cerebrospinal fluid;

2.1.2.12 More than 30% leukemic cells in blood;

2.1.2.13 Stage III WHO/ECOG score;

2.1.2.14 Organ recipients;

2.1.2.15 Pregnant or breastfeeding;

2.1.2.16 Active, uncontrolled infection, including hepatitis B, hepatitis C or HIV;

3. Phase I study of sequential chimeric antigen receptor T cell targeting at different B-cell antigens in relapsed B-cell acute lymphoblastic leukemia.

We conducted a phase I study (ClinicalTrials#: ChiCTR-OIB-17013670) of sequential chimeric antigen receptor T cell targeting at different B-cell antigens in relapsed B-cell acute lymphoblastic leukemia in Beijing Boren Hospital. The study was approved by the institutional review board of Beijing Boren Hospital, and informed consent was obtained in accordance with the Declaration of Helsinki. All these patients matched the diagnostic criteria for r/r B-ALL according to the WHO classification and completed morphological evaluation, immunophenotype analysis by FCM, cytogenetic analysis by routine G-banding karyotype analysis and leukemia fusion gene screening by RT-PCR. Patients were eligible if they were heavily treated B-ALL who failed from chemotherapy or continued MRD$^+$ after chemotherapy, and patients had positive CD19 and CD22 expression on leukemia blasts by FCM (>95%CD19 and CD22 positive). Enrolled patients received CD19 and CD22 CAR T cell infusion between January 2nd, 2018 and April 19th, 2019 and were evaluated for responses and adverse effects. After CAR T-cell

infusion, clinical outcomes including OS, DFS, adverse effects and relapse were evaluated up to date as of November 1st, 2019.

Inclusion and exclusion criteria for enrolling patients

3.1 Inclusion Criteria

3.1.1 Patients who were diagnosed as primary refractory or relapsed B-ALL1. All the patients matched the diagnostic criteria of ALL according to the WHO classification, and conducted morphological evaluation, immunophenotype analysis by flow cytometry (FCM), cytogenetic analysis by routine G-banding karyotype analysis, screen of 56 leukemia-related fusion genes by multiplex nested reverse transcriptase polymerase chain reaction (RT-PCR), and quantification of fusion genes by real-time PCR with ABL1 as reference. 339 hematological malignancies- related genes were also screened by Illumina sequencing. Extramedullary diseases (EMDs) were confirmed $CD19^+$ and $CD22^+$ by FCM and evaluated PET/CT, CT, MRI or ultrasonography. The patient relapsed during chemotherapy, failed from re-induction chemotherapy (including first and second generation TKIs) after relapse or had a persistent positive MRD for three months. Patients had positive CD19 and CD22 expression on leukemia blasts by FCM (>95% CD19 and CD22 positive);

3.1.2 Age from 1 to 18 years old;

3.1.3 Children candidates can be recruited after the legal guardian or patient advocate has signed the treatment consent form and voluntary consent form.

3.2 Exclusion criteria

3.2.1 Intercranial hypertension or unconscious;

3.2.2 Acute heart failure or severe arrhythmia;

3.2.3 Acute respiratory failure;

3.2.4 Other types of malignanttumors;

3.2.5 Diffuse intravascularcoagulation;

3.2.6 Serum creatinine and/or blood urea nitrogen over 1.5 times than normal range;

3.2.7 Sepsis or other uncontrolled infection;

3.2.8 Uncontrolled diabetesmellitus;

3.2.9 Severe psychologicaldisorder;

3.2.10 Obvious cranial lesions with cranial MRI;

3.2.11 More than 20 counts/ul leukemic cells in cerebrospinal fluid;

3.2.12 More than 30% leukemic cells in blood;

3.2.13 Stage III WHO/ECOG score;

3.2.14 Organ recipients;

3.2.15 Active, uncontrolled infection, including hepatitis B, hepatitis C or HIV;

Screening of full-human anti-CD22 scFvs from a yeast display library

The full-human CD22-specific scFvs was screened from a yeast display human scFv library (Adimab, Lebanon). The DNA sequences encoding scFvs were generated from the heavy and light chain variable regions of various human antibodies by polymerase chain reaction (PCR), and cloned into an expression

vector by homologous recombination, with the fusion to an activation domain (AD). The DNA sequence of the target peptide was cloned into another type of expression vector with fusion to a DNA binding domain (BD). A vector carrying a reporter gene with a specific DNA binding site were transduced into yeast cells. Then, the scFv-AD and Target-BD vectors were co-transformed into yeast cells[2]. If scFv-AD bound to the Target-BD, the Target-BD will bring the scFv-AD to the promoter to drive expression of the reporter gene. Yeast clones showing the reporter gene expression were selected, and the scFv sequences inside these clones were then identified.

NFAT reporter assay

The lentiviral vector pGL4.30 containing the firefly Luciferase (ffLuc) gene (Promega, Madison, U.S.), was removed the hPEST sequence, and transduced into Jurkat cells to create Jurkat-ffLuc reporter cells. The specific CD22-BBz vector was mixed at a 3:1 ratio with the pGL4.75 vector containing the synthetic Renilla Luciferase (Rluc) gene (Promega) as an internal control for transfection efficiency, and electroporated into Jurkat-ffLuc cells. After 48 hours, the CD22-BBz expression level was detected and the Jurkat-ffLuc/CD22-BBz cells were co-incubated with the same number of $CD22^{high}$ Raji, $CD22^{low}$ JVM2 cells, and $CD22^{-}$ K562 cells, or cultured in medium alone in a 96-well plate in triplicates. After an incubation for 24 hours, the cells were subjected to the measurement of luciferase activity using the Dual Luciferase Reporter Gene Assay Kit (YEASEN, Shanghai, China). Relative light unit (RLU) was calculated as the reaction enzyme value of ffLuc divided by the reaction enzyme value of RLuc. The CD22-bbz variants that elicited the greatest nuclear factor of activated T cells (NFAT) activity were selected for the subsequent analyses.

CD107a assay

Primary T cells lentivirally transduced with different CD22-BBz variants were co-cultured with $CD22^{high}$ Raji (ATCC, CCL-86), Reh (ATCC, CRL-8286), and Nalm6 (DSMZ, ACC128) cells, $CD22^{low}$ JVM-2 (Tongji Hospital, Huazhong University of Science and Technology, Wuhan, China.) cells, and $CD22^{-}$ K562 (K562 was from ATCC, CCL-243), Jurkat (ATCC, TIB-152), and U266 (ATCC, TIB-196) cells at a effector (E):target cell (T) ratio of 0.3:1 in a 96-well plate well for 3 hours with the addition of monensin (BioLegend, CA, US, Cat#420701). Cells were then harvested and stained with anti-CD8 APC (BD, CA, US, Cat#555369), anti-epidermal growth factor receptor (EGFR) Alexa Fluor 488 (BioLegend, Cat#352908), and anti-CD107a PE-Cy7 (Biolegend, Cat#328618), and analyzed by flow cytometry.

Cytotoxicity assay

The ability of specific CD22-BBz T cells to lyse target cells was analyzed using a bioluminescence assay. The $CD22^{high}$ Nalm6 and Reh cells were transduced with ffLuc as target cells. CD22-BBz T cells and ffLuc-expressing target cells were co-cultured in a 96-well plate in triplicates at effctor (E): target cell (T) ratios of 2:1, 1:1, 0.5:1 and 0:1 for 24 hours at 37°C. The cells were then subjected to luciferase assay. Percent lysis was calculated as: % specific lysis = (spontaneous death relative light unit (RLU) − test RLU)/(spontaneous death RLU) × 100.

Membrane proteome array

Membrane proteome array (MPA) was conducted at Integral Molecular, Inc. (Philadelphia, U.S.). The MPA is a protein library composed of 5,300 distinct human membrane protein clones, each overexpressed in live cells with expression plasmids. Each clone was individually transfected in separate wells of a 384-well plate followed by a 36 hour incubation[3]. Cell clones expressing individual membrane protein were arrayed in duplicates with a matrix format for high throughput flow cytometric screening. Before screening, anti-CD22 scfv concentration for screening was determined on cells expressing positive (membrane-tethered CD22) and negative (mock- transfected) binding controls, followed by detection with flow cytometry using a fluorescently- labeled secondary antibody. Anti-CD22 scfv was added to the MPA at the predetermined concentration, and binding across the protein library was measured on the Intellicyt iQue using a fluorescently-labeled secondary antibody. Each array plate contains both positive (CD22) and negative (mock) controls to ensure plate-by-plate reproducibility.

Xenograft model and bioluminescence imaging

Animal sample size was decided according to International Council on Harmonisation guidelines. no statistical methods were used to estimate sample size. All animal studies were approved by the Institutional Animal Care and Use Committee (IACUC) of Nanjing IASO Biotherapeutics (ACU19-896). NOD-Cg.Prkdc^{SCID}IL-2Rgc^{null}/vst (NPG) mice (female, aged 4–5 weeks) were obtained from Beijing Vitalstar Biotechnology Co., Ltd. and raised under pathogen-free conditions. Mice were implanted with 1×10^6 Nalm6 cells that were engineered to express luciferase (Nalm6-Luc) by tail-vein injection followed by intravenous infusion of different doses (5×10^5 and 2×10^6) of CD22-CAR T cells and mock-transduced T cells 2 days later. Recipients with similar tumor burdens were randomly distributed evenly across the groups prior to T cell infusion. No blinding to the group allocation during the experiment. To monitor tumor burden, mice received intraperitoneal luciferin D injection and were imaged using Bruker imaging system at indicated time points. Photon emission from Nalm6-Luc in mice was indicated as photon per second per cm^2 per steradian. Mice were euthanized at the end of the experiment (day 28) according to the Protection of Animals Act.

Clinical trial design and patient enrollment

A phase I trial of CD22-CAR^{FH80} T cell therapy was conducted in 8 r/r B-ALL pediatric patients who failed in prior humanized CD22-CAR^{YK-002} therapy, aged 5-12 years, between January 9, 2020 and July 16, 2020, at Beijing Boren Hospital. The study was approved by the institutional review board of Beijing Boren Hospital, and informed consent was obtained from individual patient in accordance with the Declaration of Helsinki. The trial was registered on Chinese Clinical Trial Registry/WHO International Clinical Trial Registry (ChiCTR2000028793). Patients were eligible if they were CD22$^+$ B-ALL and failed prior CD19 and CD22 CAR T therapies and the details of inclusion and exclusion (I/E) criteria are shown in Supplementary Methods. The baseline disease status was assessed immediately before enrollment (Details in Table S2). After patient enrollment, there was no further bridging chemotherapy beforelymphodepleting procedure.

Manufacture and infusion of CAR T cells

CD22-CAR^{FH80} T cells were manufactured with T cells, obtained through leukapheresis, and transduced with a lentiviral vector expressing the CD22-CAR^{FH80}, with the same protocol as our prior production of CD19-

CAR T cells[4]. Peripheral blood mononuclear cells collected from patients or donors were stimulated with magnetic beads coated with anti-CD3/CD28 antibodies (Life Technologies, Carlsbad, CA, USA) overnight. The next day, transduction was performed at a multiplicity of infection ratio of 1:10. Transduced cells were cultured in X-VIVO 15, a serum-free medium (Lonza) with 300 IU/ml interleukin-2. Transduction efficiency (as the percentage of CAR^+ cell among $CD3^+$ cells) and cell viability were determined immediately before cell infusion by flow cytometry and Trypan blue exclusion respectively.

After leukapheresis, patients underwent lymphodepletion with Fludarabine at 30 mg/m^2/day and Cyclophosphamide at 250 mg/m^2/day, from day -5 to -3 before CD22-CAR^{FH80} T cell infusion (day 0). CAR T cells were infused at a single time point. When the harvest of CD22-CAR T cells was less than 0.1×10^5 per kg patient weight, we defined it as a manufacture failure. The maximum infused dose of CAR T cells was 10 $\times 10^6$ per kg patient weight.

Response assessment

All patients underwent bone marrow (BM) biopsy and radiological examination on day 30 to determine the response and remission status. Complete remission (CR) or CR with incomplete count recovery (CR/CRi), relapse and minimal residual disease (MRD) were defined in accordance with the National Comprehensive Cancer Network (NCCN) guidelines, version 1.2020. MRD^- was defined as the absence of leukemia cells in BM determined by flow cytometry. The sensitivity of the MRD analyses was 0.01%.

Assessment and management of adverse events

Cytokine-release syndrome (CRS) and immune effector cell-associated neurotoxicity syndrome (ICANS) were graded by the most severe event according to ASTCT Consensus[5]. Specific organ toxicities were graded according to the National Cancer Institute Common Terminology Criteria for Adverse Events, CTCAE Version 5.0. Methylprednisolone (2 to 15 mg/kg/d, intravenous injection (IV)) were given in patients with grade ≥ 3 CRS. Mannitol (2.5 ml/kg/d, IV), furosemide (1 mg/kg/d, IV) and dexamethasone (2-5 mg/d, intrathecal injection) were used in patients with grade ≥ 2 neurotoxicity. The details of the managements of CRS and ICANS and other supportive cares are in Supplemental Methods.

Statistical analysis

Difference between two groups was analyzed by two-tailed, unpaired two-sample t-test. All statistical analyses were performed using SPSS Statistics version 26, and P values of < 0.05 were considered significant.

Supplemental Results

CD22-CAR^{YK002} T-cell second treatment was ineffective

Four B-ALL patients (A-D) who relapsed from CD22-CAR^{YK002} therapies had received a second infusion of CD22-CAR^{YK002} T cells (patient A, B were enrolled in ChiCTR-OIC-17013523; patient C, D were enrolled in ChiCTR-OIB-17013670) between May 8, 2018 and April 29, 2019 at Beijing Boren Hospital. The characteristics of these patients were shown in Table S1. The treatment history of these patients was illustrated in Fig. S1a. CD22-CAR^{YK002} T cells were manufactured as previously described.[4] After leukapheresis, patients received lymphodepleting chemotherapy before CD22 CAR^{YK002} T-cell infusion (day 0). The detailed infused dose of CD22-CAR^{YK002} T cells was detailed in Table S1. Patients B, C and D displayed no response to the second infusion of CD22-CAR^{YK002} T cells; patient A had a transient complete response but quickly relapsed. The secondarily infused CD22-CAR^{YK002} T cells failed to expand as assessed by flow cytometry in all patients (Fig. S1b). The treatment overview was illustrated in Fig. S1a. These results collectively indicated that CD22-CAR^{YK002} second infusion were ineffective in patients who relapsed after primary CD22-CAR^{YK002} therapies.

Development of a novel full-human CD22-CARFH80 with superior activity

Full-human anti-CD22 scFvs were screened from a full-human scFv yeast display library. The detailed procedure was described in supplemental methods. To create a panel of CD22-BBz variants that harbored different anti-CD22 scFv fragments, the screened anti-CD22 scFvs were fused to the intracellular 4-1BB co-stimulatory and CD3ζ signaling domains, and further linked to epidermal growth factor receptor (tEGFR) with Thoseaasigna virus 2A (T2A) to facilitate detection of CAR and elimination of CAR T cells when necessary (Fig. 1a). We next test the activation of Jurkat T cells transiently transduced with different CD22-BBz variants, in response to CD22^{high} Raji, CD22^{low} JVM-2 and CD22^{-} K562 cells (Fig. 1b) with NFAT reporter assay. The results showed that T cells transduced with CD22-BBz 80, 27, 36, 6 and 43 had the highest NFAT activation when co-cultured with Raji cells. However, CD22-BBz 6 and 43 also elicited marked NFAT activation in T cells without co-culturing with leukemia cells, probably owing to the off-target recognition or tonic signaling, and they were therefore excluded from further analyses. CD22-BBz 51, 15 and 23 had the low NFAT activation when co-cultured with Raji cells. Markedly. CD22-BBz 80, 27 and 36 also elicited substantial NFAT activation when co-cultured with JVM-2 cells, suggesting that these variants could triggers signaling even in response to target cells with low level of CD22 expression (Fig. 1c and Fig. S2). CD22-BBz 80, 27 and 36 were thus defined as constructs which could transmit strong antigen-specific activation signals in T cells.

We then evaluated the effector function of primary T cells lentivirally transduced with CD22-BBz variants 80, 27, 36 and 51 (as a low NFAT activity control) via CD107a degranulation and cytotoxicity assay. High proportions of CD107a positivity (>30%) were detected in T cells bearing CD22-BBz 80, 27 and 36 when co-cultured with CD22high Raji, Reh and Nalm6 cells. When co-cultured with the CD22low JVM-2 cells, the CD107a expression was higher in T cell bearing CD22-BBz 80 and 36 than that bearing CD22-BBz 27. However, T cells bearing CD22-BBz 36 also showed considerable proportions of CD107a positivity when co-culturing with CD22- K562, Jurkart and U266 cells and in medium alone, indicating nonspecific off-target

effects. Lower proportions of CD107a positivity (<15%) were detected in T cells bearing CD22-BBz 51 when co-cultured with Reh, Nalm6 and JVM-2 cells. Three independent experiments from three different donors have been conducted with similar results (Fig. 1d). Cytotoxic assay indicated that T cells transduced with CD22-BBz 80 produced a slightly stronger cytolytic activity than CD22-BBz 27 and 36 T cells when co-cultured with Nalm6 and Reh cells (Fig. 1e). However, none of these CD22-BBz variants mediated obvious cytolytic activity against the JVM-2 cells, probably owing to a very refractory nature of JVM-2 cells, or the too low expression level of CD22 on JVM-2 cells. Nevertheless, CD22-BBz 80 was identified as a construct capable of eliciting the greatest T cell effector activity against target cells.

In concordance with the lack of cytolytic effect against the CD22- cell lines, the membrane proteome array (MPA) showed that CD22-bbz 80 had a high specificity to the target antigen, suggesting a minimal risk of off-target effect if applied in therapy. (Fig. 1f). To confirm the anti-leukemia effect of CD22-BBz 80 T cells in vivo, NOD-Cg.Prkdc^{SCID}IL-2Rgc^{null}/vst (NPG) mice were injected with 1×10^6 Nalm6-Luc cells 2 days before the treatment with different doses (0.5 and 2×10^6) of CD22-BBz 80 T cells and mock-transduced T cells. At the higher dose, CD22-BBz 80 T cells eliminated the Nalm6 tumors in two of the three mice treated, whereas at the lower dose, the tumor growth was significantly retarded despite that the tumor cells could not be completely eliminated (Fig. S3). In contrast, mock-transduced T cells were ineffective against tumor growth. Thus, the full-human CD22-BBz 80 construct, which could mediate a potent and antigen-specific anti-leukemia activity, was termed CD22-CARFH80 thereafter and used in the subsequent clinical study.

Supplemental Table 1. Baseline Characteristics of Patients Treated with CD22-CAR^{YK002} T cells

	Demographics		Prior therapy			Status					
Pt. No.	Sex	Age (years)	HSCT	CD19 Immunotherapy times§	CD22 Immunotherapy times§	Bone marrow blasts by morphology (%)	Bone marrow blasts by FCM (%)	Extramedullary disease	Blast CD19 Expression	Blast CD22 Expression	Total CAR T cells infused ($x10^6$/kg)
A	F	10	Y	1	1	25	16.18	---	Neg	Pos	0.50
B	M	6	N	1	1	93	79.55	---	Pos	Pos	2.06
C	M	4	N	1	1	81.50	55	CNSL	Pos	Pos	5.80
D	F	10	N	1	1	98	90.42	Anterior superior mediastinum	Neg	Pos	1.60

Pt, patient; M, male; F, female; HSCT, allogeneic hematopoietic stem cell transplantation; Y, yes; N, no; FCM, flow cytometry; CNSL, central nervous symptom leukemia; Neg, negative; Pos, positive; [§]the detailed response and progress of CAR T therapy was shown in supplementary figure 1.

Supplemental Table 2. Baseline Characteristics of Patients Treated with CD22-CAR^{FH80} T cells

	Demographics		Genetic Aberrations			Prior therapy			Status					
Pt. No.	Sex	Age (years)	Gene mutation	Gene fusion	Complex chromosome	HSCT	CD19 Immunotherapy times§	CD22 Immunotherapy times§	Bone marrow blasts by morphology (%)	Bone marrow blasts by FCM (%)	Extramedullary disease	Blast CD19 Expression	Blast CD22 Expression	CD22 MFI before CD22-CAR^{FH80}
1	F	16	N	E2A-PBX1	N	Y	1	1	-	0.06	---	Pos	Pos	3692
2	F	7	KRAS, TP53	N	N	N	2	1	97	94.37	---	Neg	Pos	3695
3	M	6	IKZF1	N	46,XY,del(2)(p15),?del(8)(p21),inc(cp4)/46,XY(1)	N	1	2	90	43.13	---	Pos	Dim	113
4	M	7	N	N	N	Y	1	2	52	28.74	---	Pos	Dim	106
5	F	5	KRAS	N	53-54,XX,+X,+4,+6,+17,+21,+21,+22[CP10]	N	2	1	11	8.44	---	Pos	Pos	4880
6	F	10	TET2	N	47,XX,t(2;14)(q33;q13),+8[8]/47,idem,t(5;8)(q31;p21)[6]/45,X,-X,t(2;14)(q33;q13),t(9;15)(p11;q26)[5]/46,XX[1]	Y	3	2	-	-	Right side posterior eyeball, chest wall, scapula, posterior sternum, pleura, hilum and accessory area	Pos	Pos	-
7	F	11	IKZF1, ETV6, FLT3, EZH2, PAX	N	N	N	1	1	50	25	---	Pos	Pos	10190
8	F	12	JAK1, KRAS	E2A-HLF	N	Y (2)	1	1	16	1.26	---	Pos	Pos	9272

Pt, patient; M, male; F, female; HSCT, allogeneic hematopoietic stem cell transplantation; Y, yes; N, no; FCM, flow cytometry; MFI,Mean Fluorescence Intensity; Neg, negative; Pos, positive; Dim, diminish; §the detailed response and progress of CAR T therapy was shown in supplemental figure 1.

Supplementary Table 3. Characteristics of Infused CD22-CAR^{FH80} T Cell Product

Pt. No.	Lymphodepleting Chemotherapy	Cell Dose (x10^6/kg)	% Transduced (Protein L)	Cell Viability (%)	Cryopreserved Prior to Infusion
1	Flu (30 mg/m^2/day) and Cy (250 mg/m^2/day)	1.43	56.4	75.1	No
2	Flu (30 mg/m^2/day) and Cy (250 mg/m^2/day)	0.96	54.3	76.5	No
3	Flu (30 mg/m^2/day) and Cy (250 mg/m^2/day)	2.5	37.07	81	No
4	Flu (30 mg/m^2/day) and Cy (250 mg/m^2/day)	1	57	89.1	No
5	Flu (30 mg/m^2/day) and Cy (250 mg/m^2/day)	3.49	44	76.5	No
6	Flu (30 mg/m^2/day) and Cy (250 mg/m^2/day)	9.4	39.3	87.1	No
7	Flu (30 mg/m^2/day) and Cy (250 mg/m^2/day)	0.68	27.7	80.9	No
8	Flu (30 mg/m^2/day) and Cy (250 mg/m^2/day)	0.2	30.1	75.3	No

Pt, patient; Flu, Fludarabine; Cy, Cyclophosphamide.

Supplemental Table 4. The Characteristics and Donor Types of Patients Who Received HSCT after CD22-CAR^{FH80} T Therapy

	Pt01	Pt03	Pt07
**Donor types**	Haploidentical donor	Haploidentical donor	Haploidentical donor
**Stem cell sources**	Peripheral blood	Peripheral blood	Peripheral blood
**Conditioning regimens**	TBI/CY/Ara-C/Flu/ATG	TBI/CY/Ara-C/Flu/ATG	TBI/CY/Ara-C/Flu/ATG

TBI, total body irradiation; Bu, busulfan; CY, cyclophosphamide; Ara-C, cytarabine; Flu, fludarabine; ATG, anti-thymocyte globulin.

Supplemental Table *5*. CD22-CAR^{FH80} T Cell Expansion, Clinical Response and Toxicity

Pt. No.	CAR T Cell Expansion					Toxicity		Response	
	Maximum Circulating CAR (×106/L)	Maximum Circulating CAR %#	Marrow$ (%)	CSF$ (%)	Pleural Fluis$ (%)	CRS Grade	ICANS Grade	Maximum Response	Response Duration
1	10.3	14.27	---	-	---	Grade 1	-	CR£+	11 mos+
2	19.8	16.97	---	-	---	Grade 1	-	CR£	6 mos R§
3	16.8	-	---	6.98	---	Grade 1	Grade 2	CR£	4 mos R
4	408	86.45	---	0	---	Grade 1	-	CR£+	9 mos+
5	1.01	1.00	---	---	---	-	-	NR	NR
6	308	32.47	---	32.00	---	Grade 1	-	PR**	2 mos¢
7	73.5	78.85	---	---	---	Grade 3	Grade 3	CR£+	6 mos+
8	19.8	14.27	---	---	---	Grade 1	-	CR£+	5 mos+

Pt, patient; CRS, Cytokine Release Syndrome; ICANS, immune effector cell-associated neurotoxicity syndrome; CR, complete response; NR, no response; PR, partial response; R, relapse. #% of CD3 cells co-expressing CD22-CAR; $% of T cells on Day 28±4; £Minimal residual disease (MRD) negative bone marrow, **Attained partial response in the EMDs on Day 30, continued to response until day 40. +Response ongoing, ¢death due to sepsis, ¢death due to sepsis, §death due to tumor progress.

Supplemental Table 6. Adverse Events Summary During CD22-CAR^{FH80} T Cell Therapy

	Grade 0 (%)	Grade 1 (%)	Grade 2 (%)	Grade 3 (%)	Grade 4 (%)	Grade 5 (%)
	Number of patients (percent)					
**CRS**						
Fever	1 (13)	7 (88)	-	-	-	-
Hypoxia	7 (88)	-	-	1 (13)	-	-
Hypotension	8 (100)	-	-	-	-	-
**ICANS**						
ICE score	8 (100)	-	-	-	-	-
Depressed level of consciousness	7 (88)	-	1 (13)	-	-	-
Seizure	7 (88)	-	-	1 (13)	-	-
Motor weakness	8 (100)	-	-	-	-	-
Elevated ICP/cerebral edema	8 (100)	-	-	1 (13)	-	-
**Blood & Lymphatic System Disorders**						
Intracranial hemorrhage	8 (100)	-	-	-	-	-
Gastrointestinal hemorrhage	8 (100)	-	-	-	-	-
**Gastrointestinal Disorders**						
Constipation	8 (100)	-	-	-	-	-
Diarrhea	8 (100)	-	-	-	-	-
**Infections and Infestations**						
Catheter related infections	8 (100)	-	-	-	-	-
Sepsis	7 (88)	-	-	-	-	1 (13)
**Metabolism and nutrition disorders**						
Hypertriglyceridemia	8 (100)	-	-	-	-	-
Hypokalemia	8 (100)	-	-	-	-	-
Hyponatremia	8 (100)	-	-	-	-	-
Hypophosphatemia	8 (100)	-	-	-	-	-
**Respiratory, thoracic and mediastinal disorders**						
Epistaxis	8 (100)	-	-	-	-	-
**Vascular disorders**						
Hypertension	7 (88)	1 (13)	-	-	-	-
**Cardiac disorders**						
Sinus tachycardia	8 (100)	-	-	-	-	-

CRS and ICANS were grading according to the American Society for Transplantation and Cellular Therapy (ASTCT). Adverse organ reaction was grading according to CTCAE v5.0. Abbreviations ICP, intracranial pressure, ICE score, immune effector cell-associated encephalopathy score, CRS, cytokine release symptom, ICANS, immune effector cell-associated neurotoxicity syndrome.

Figure S1. CD22-CAR^{YK002} T-cell second treatment was ineffective in 4 patients who relapsed after primary CD22-CAR^{YK002} therapies

a, Swimmer plot illustrating the treatment history of 4 patients relapsed from prior therapy with CD22-CAR^{YK002} T cells, and the clinical response after a second CD22-CAR^{YK002} T cells re-infusion. Patient A achieved complete remission after second infusion of CD22-CAR^{YK002} T cells, but she relapsed after one month; patient B and C had not response to second CD22-CAR^{YK002} T cells infusion; patient D achieved minimal residual disease-positive remission in response to second CD22-CAR^{YK002} T cells infusion at day 15, but soon relapsed at day 30. HSCT, hematopoietic stem cell transplantation; MRD, minimal residual disease. b, Absolute numbers of CAR T cells detected by flow cytometry in the peripheral blood from two patients after the first and second infusion of CD22-CAR^{YK002} T cells. No patients exhibited no detectable expansion of secondly infused CD22-CAR^{YK002} T cells, as assessed by flow cytometry.

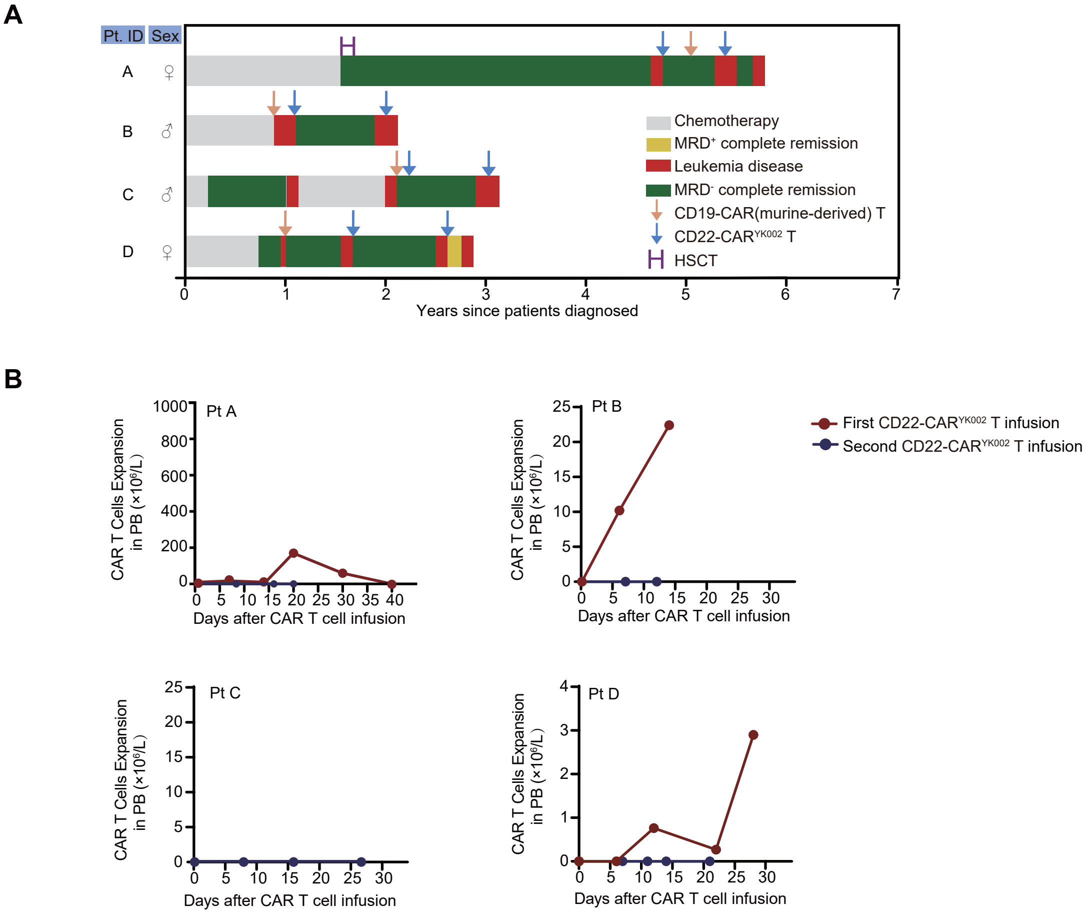

Figure S2. Nuclear Factor of Activated T cells (NFAT) Reporter Gene Assay
NFAT reporter gene assay in T cells transduced with different CD22-BBz variants after co-incubating with CD22-high target cells (Raji), CD22-low target cells (JVM-2), CD22-negative cells (K562), or with medium alone.

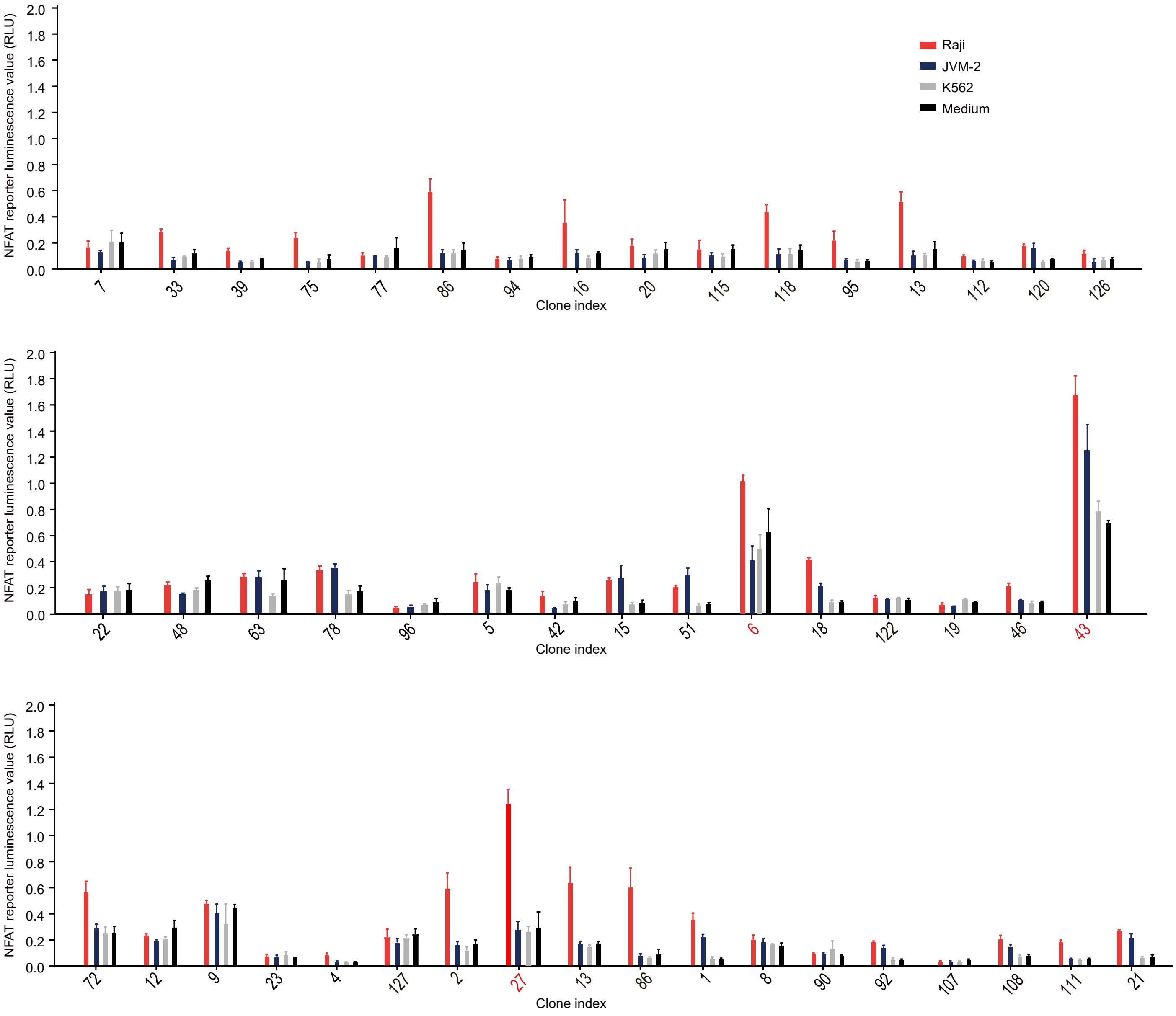

Figure S3. CD22-CAR^{FH80} T Cells show superior anti-leukemia activity in vivo
Xenograft model demonstrating the in vivo activity of CD22-CAR^{FH80} T cells at indicated doses or mock-transduced T cells intravenously administered 2 days after Nalm6-Luc cell implantation. Tumor growth in each mouse was evaluated by measuring the photon using Bruker imaging system. The data are representative of at least 2 independent experiments with similar results.

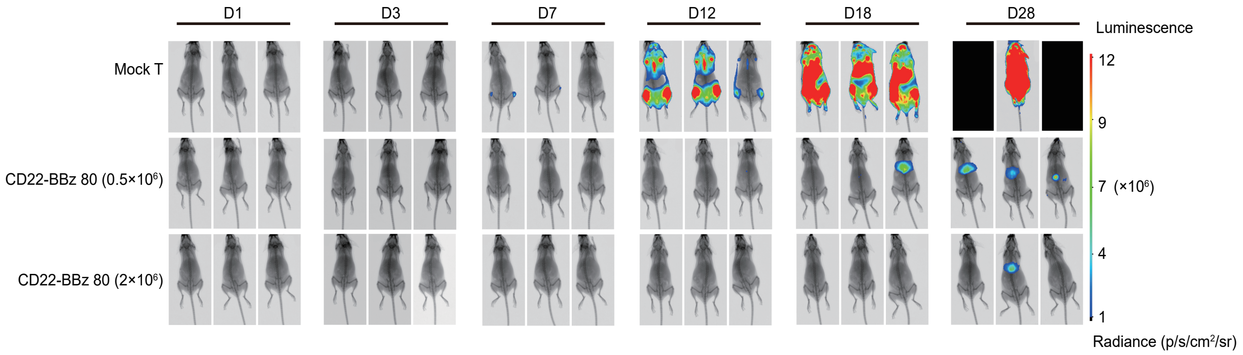

Figure S4. Treatment history in 8 patients before enrolling in CD22-CAR^{FH80} therapy
Swimmer plot illustrating the treatment history of CD22-CARFH80 T clinical trial enrolled patients with B-ALL. Arrows indicate CAR T therapy. Patient number are shown to the left of the y axis. MRD, minimal residual disease; HSCT, allogeneic hematopoietic stem cell transplantation.

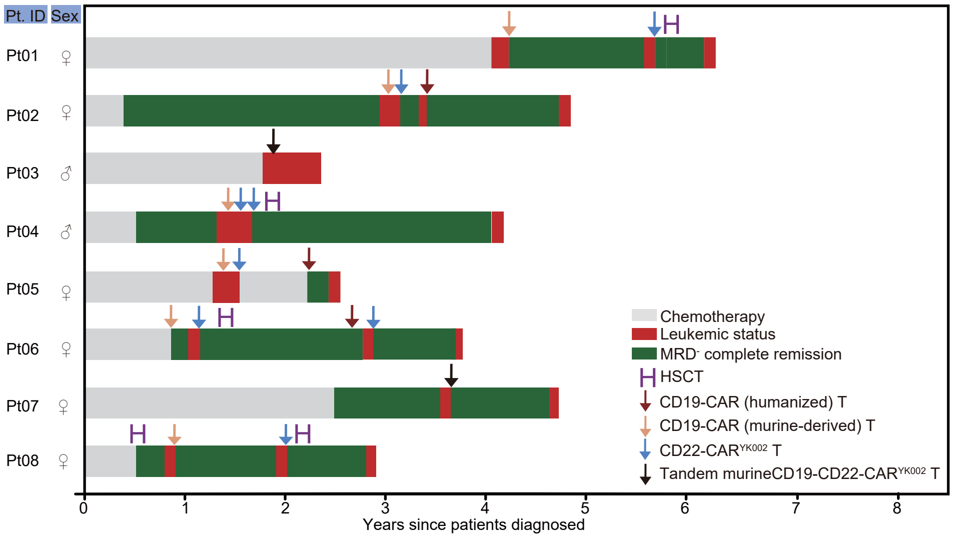

Figure S5. MRI Scans of Intracranial Infection in Pt 06

MRI scans showing diffused intracranial infection in Pt 06 at day 41 after CD22-CAR^{FH80} T cells infused.

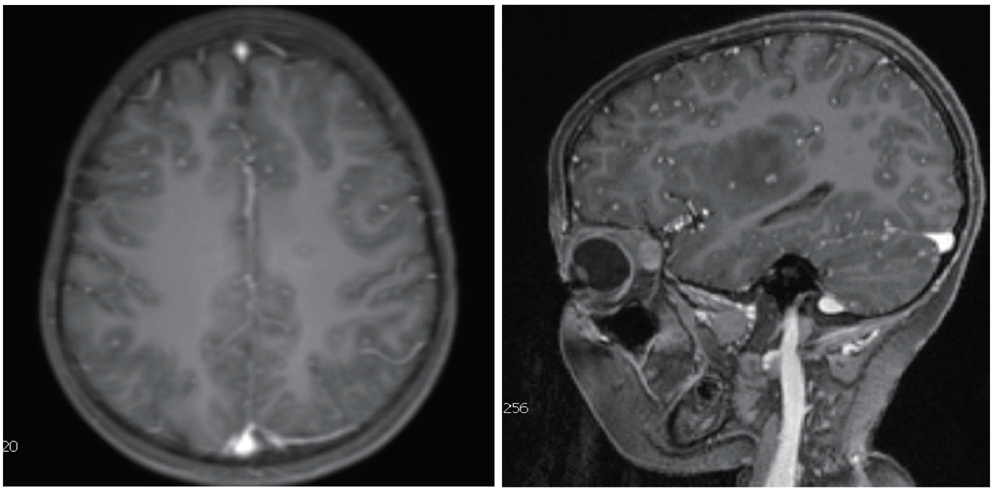

Figure S6. Clinical response and change of CD22 expression in response to CD22-CAR^{FH80} T cell therapy

Dot plots showing the proportions of blasts (red) and non-tumor cells (black) among all mononuclear cells from patients at indicated time points, and contour plots showing CD22 and CD19 expression on blasts (red) before and after CD22-CAR^{FH80} T cell therapy, determined by flow cytometry with a population of CD22-negative non-B cells with similar cell size in the same staining tube (blue) as negative control for evaluating CD22 expression level. The blasts were defined based on the combined analysis of multiple makers. The numbers in the plot indicate the proportion of blasts in the four quadrants.

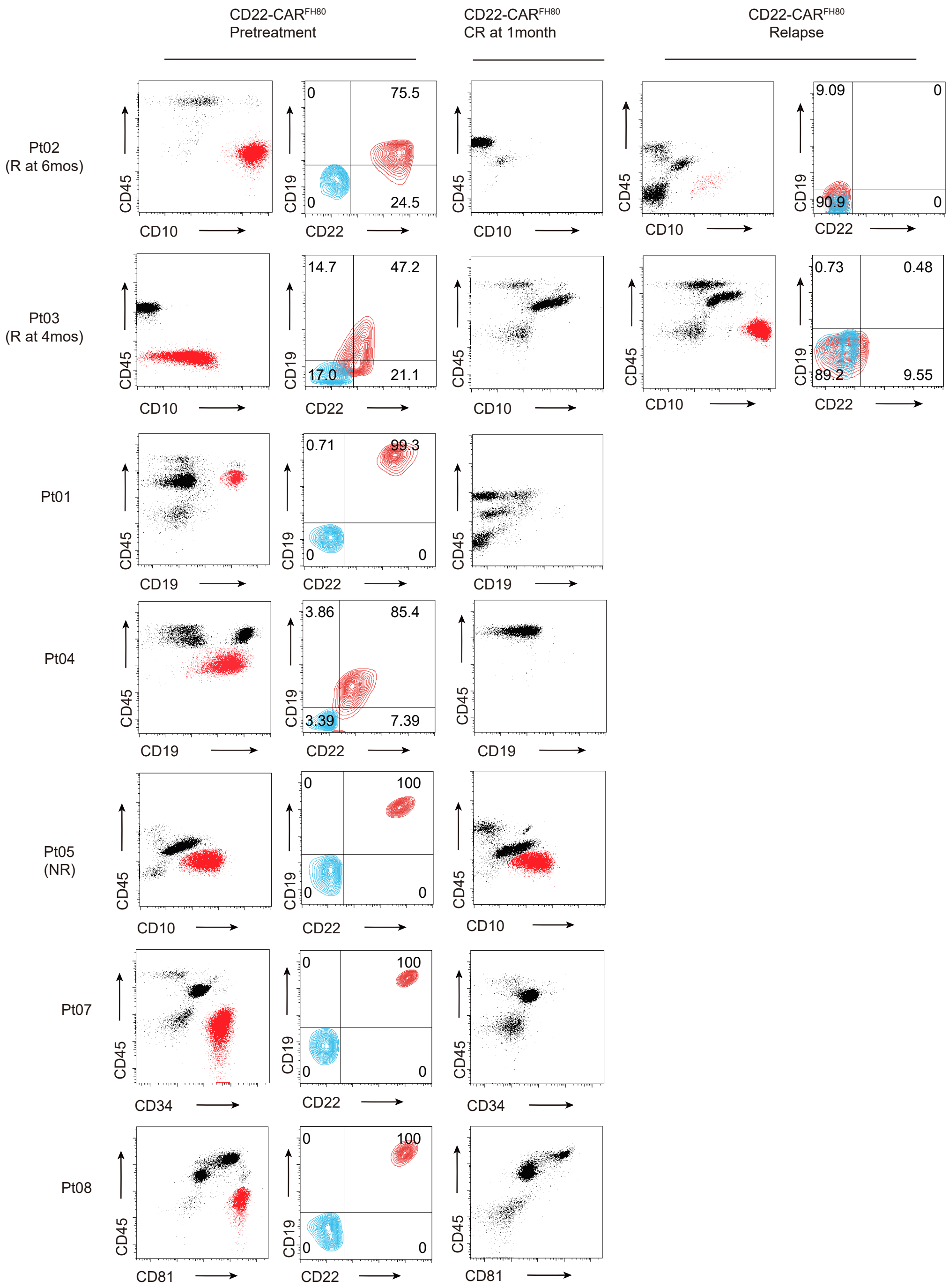

Figure S7. The CRS and ICANS Grade Comparison between CD22-CAR^{FH80} and CD22-CAR^{YK002} Therapy

The comparison of the CRS and ICANS grade between CD22-CAR^{FH80} and CD22-CAR^{YK002} CAR T therapy in the same patient. *P* values were calculated by two-tailed Wilcoxon matched-pairs signed-rank test.

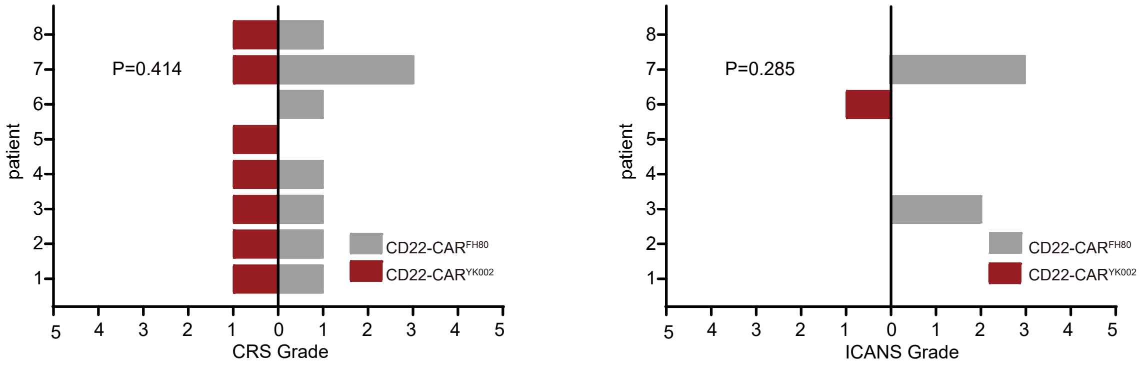

Figure S8. Elevation in serum cytokine levels after CD22-CARFH80 T cell infusion
Kinetics of serum cytokines indicative of systemic inflammation in individual patients after CAR T cell infusion, as determined by Quantikine enzyme-linked immunosorbent or chemiluminescence microparticle immunoassay. Dotted horizontal lines indicate normal upper limit for specific cytokines.

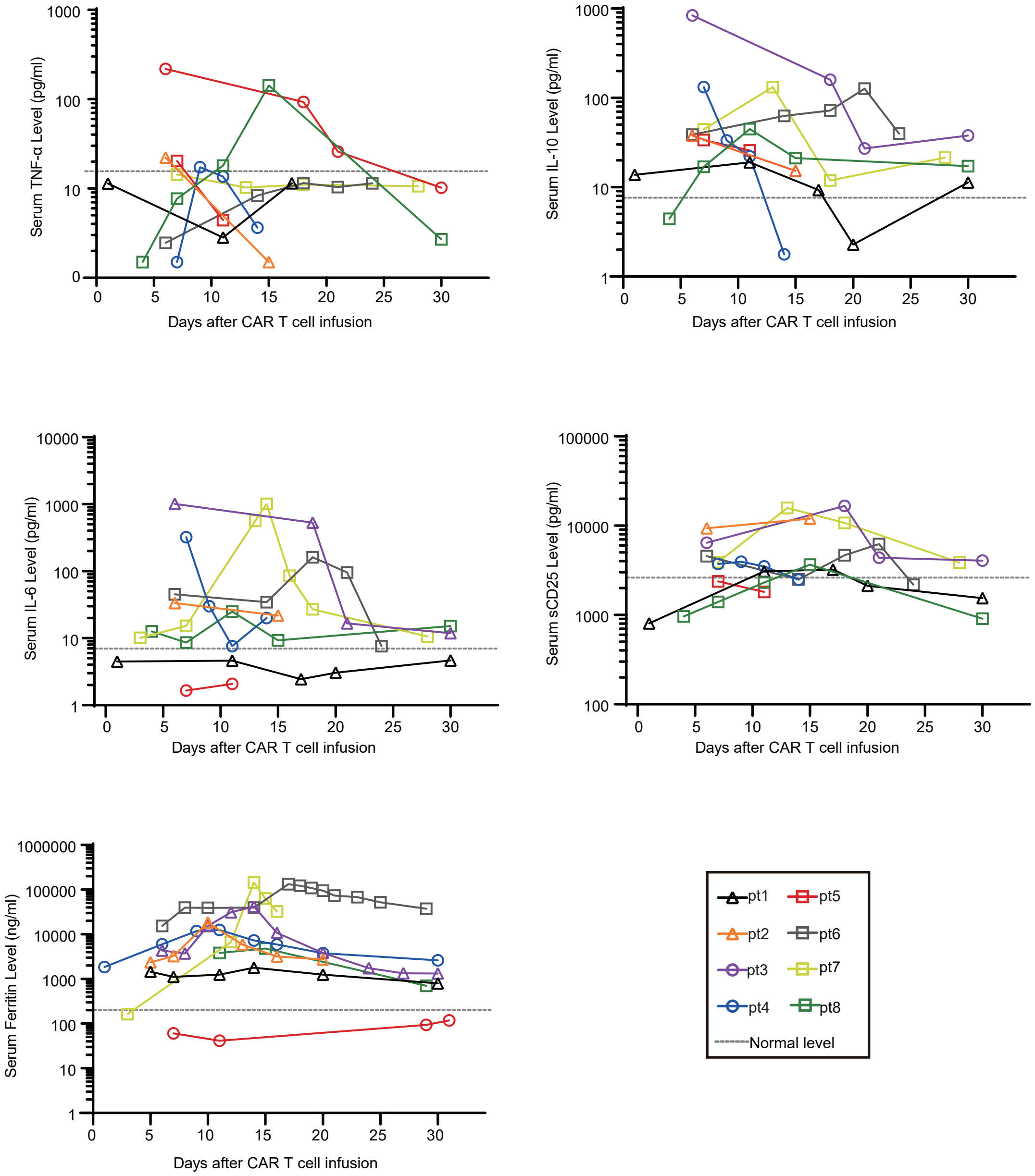

References

1. Brown, P. et al. Pediatric Acute Lymphoblastic Leukemia, Version 2.2020, NCCN Clinical Practice Guidelines in Oncology. *J Natl Compr Canc Netw.* **18,** 81-112 (2020).
2. Gietz, D. et al. Improved method for high efficiency transformation of intanct yeast cells. *Nucleic Acids Res*. **20**, 1425 (1992).
3. Tucker, DF. et al. Isolation of state-dependent monoclonal antibodies against the 12-transmembrane domain glucose transporter 4 using virus-like particles. *Proc Natl Acad Sci.* **115**, 4990-4999 (2018).
4. Pan, J.et al. CD22 CAR T-cell therapy in refractory or relapsed B acute lymphoblastic leukemia. *Leukemia.* **33,** 2854-2866 (2019).
5. Lee, DW. et al. ASTCT Consensus Grading for Cytokine Release Syndrome and Neurologic Toxicity Associated with Immune Effector Cells. *Biol Blood Marrow Transplant.* **25**, 625-638 (2019).
